# Novel immune cell phenotypes in spondyloarthritis pathogenesis

**DOI:** 10.1007/s00281-021-00837-0

**Published:** 2021-02-10

**Authors:** Daniele Mauro, Davide Simone, Laura Bucci, Francesco Ciccia

**Affiliations:** 1 Dipartimento di Medicina di Precisione, Section of Rheumatology, Università degli Studi della Campania L. Vanvitelli, Naples, Italy; 2 Nuffield Department of Orthopaedics, Rheumatology and Musculoskeletal Sciences, University of Oxford, Oxford, UK

**Keywords:** SpA pathogenesis, Cellular players, Stromal cells, Innate immunity, Adaptive immunity

## Abstract

Spondyloarthritis (SpA) is a heterogeneous group of chronic inflammatory diseases of unknown etiology. Over time, the plethora of cellular elements involved in its pathogenesis has progressively enriched together with the definition of specific cytokine pathways. Recent evidence suggests the involvement of new cellular mediators of inflammation in the pathogenesis of SpA or new subgroups of known cellular mediators. The research in this sense is ongoing, and it is clear that this challenge aimed at identifying new cellular actors involved in the perpetuation of the inflammatory process in AxSpA is not a mere academic exercise but rather aims to define a clear cellular hierarchy. Such a definition could pave the way for new targeted therapies, which could interfere with the inflammatory process and specific pathways that trigger immune system dysregulation and stromal cell activity, ultimately leading to significant control of the inflammation and new bone formation in a significant number of patients. In this review, we will describe the recent advances in terms of new cellular actors involved in the pathogenesis of SpA, focusing our attention on stromal cells and innate and adaptive immunity cells.

## Introduction

Spondyloarthritis (SpA) is a heterogeneous group of systemic inflammatory diseases divided, considering the prevalent joint involvement, into mainly axial (AxSpA) and mainly peripheral (pSpA) arthritis [1]. The pathological hallmark of SpA is the aberrant ossification that occurs in the sacroiliac joints, intervertebral discs, and entheses [2].

Although the pathogenesis of SpA is not yet fully understood, it has been shown that, along with genetic and environmental factors, different cytokines such as tumor necrosis factor-alpha (TNFα) and the IL-23/IL-17 axis could mediate the dysregulation of immune and stromal cells, leading to the imbalance between bone resorption and new tissue bone formation [3]. The clinical efficacy of anti-TNFα further confirms the link between the inflammatory milieu, immune cell activation, stromal cell involvement, and bone remodeling by controlling inflammation and partially halting X-ray damage progression [4, 5].

Nevertheless, although the consequences of uncontrolled inflammation are relatively clear, there is still debate on the hierarchical definition of the cellular elements that play a prominent role in SpA pathogenesis. In addition, large areas of uncertainty remain, for example, on which cell is the primary source of IL-17 in AxSpA and consequently of which polarizing/stimulating cytokines are ultimately more relevant in the production of IL-17 [6]. Even though both TNFα and IL-17 are valuable therapeutic targets in SpA, a significant proportion of patients still fail to respond, and particularly in AxSpA, the bone remodeling causing the radiological progression and long-term disability is not fully controlled by the current therapies.

Recent research explores the involvement of new cellular mediators of inflammation in the pathogenesis of SpA and new subsets of known cellular players. This challenge is not a mere academic exercise but rather aims to define a clear cellular hierarchy. Such a definition could pave the way for new targeted therapies, which could interfere not only with the inflammatory process but also with specific pathways that trigger immune system dysregulation and stromal cell activity possibly involved in both inflammation and bone remodeling.

**Fig. 1:**
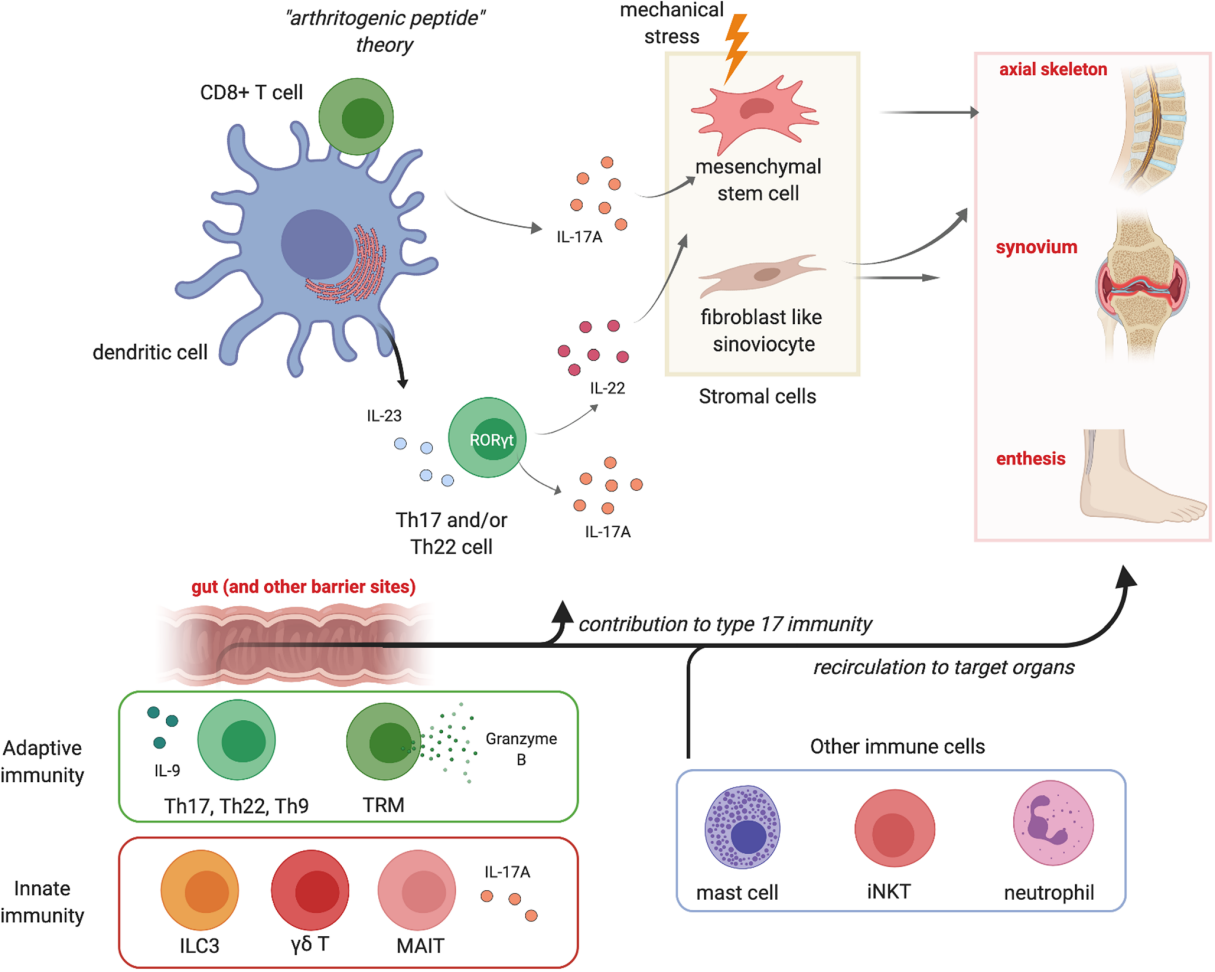
Cellular lineages and phenotypes involved in the pathogenesis of spondyloarthritis

This review aims to analyze new cellular mechanisms, in the context of stromal cells and adaptive and innate immunity, involved in the pathogenesis of SpA as summarized in Fig. 1.

## Stromal cells

The two most striking biological manifestations of SpA are the immune and non-immune mediated inflammation and the exaggerated ossification, which accounts for most of the long-term disability observed in SpA. In looking at the novel cellular players mediating the perpetuation of inflammation in SpA, it seems relevant to consider the stromal cells interacting with the immune cells. Multiple lines of evidence recognize stromal cells among the principal effectors of the structural damage in SpA. This section will briefly summarize the most relevant evidence on mesenchymal stromal cells’ involvement in SpA pathogenesis and analyze the recent data on fibroblast-like synovial cells from a therapeutic perspective.

### Mesenchymal stromal cells

Stem cells, hematopoietic and stromal, are multipotent cells capable of self-renewal. Hematopoietic stem cells (HSCs) persist in specialized niches of the bone marrow (BM) and include various cell types such as osteoblasts, endothelial cells, and BM mesenchymal stem cells (BM-MSC). BM-MSCs can perform variable functions: create a microenvironment involved in the maintenance of the HSCs [7], immunomodulation [8], differentiate in osteoblasts, adipocytes, and chondroblasts [9].

Enthesitis is a well-defined hallmark of SpA. Since entheses are closely associated with the adjacent bone marrow, it is conceivable that populations of BM cells may also invade the enthesis’ soft tissues through holes in the subchondral bony plate [10]. In this sense, it has been shown that most entheses have small holes in the bone cortex (100–400 μm wide) [10], through which smaller MSCs could migrate. Given its contiguity with the BM, it seems reasonable to imagine that the most likely source of inflammatory cells observed in entheses is indeed the BM, of which edema represents the diagnostic hallmark of axial SpA on magnetic resonance imaging (MRI).

During the last years, research in SpA has paid attention to the crucial role of mesenchymal stromal cells (MSCs) that have been found to influence irregular ossification [8]. It has been shown that BM-MSCs obtained from AS patients (AS-MSC) have a higher capacity to differentiate in osteoblasts compared to BM-MSCs obtained from healthy donors (HD-MSC) [11]. This process is mediated by the AS-MSC BMP2 overexpression, which excessively activated the Smad1/5/8 and ERK signaling pathways, and downregulation of Noggin, finally resulting in the activation of BMP pathway and consequent new bone formation [11, 12]. In this regard, IL-22, a pro-inflammatory cytokine belonging to the IL-10 family, regulated by IL-23, could play a key role in the proliferation, migration, and osteogenic differentiation of MSCs. In the study by El-Zayadi et al. [13], the combined treatment of MSC with IL-22, IFN-γ, and TNFα resulted in increased MSC proliferation and migration. IL-22 alone was unable to mediate any of the previously described effects, while it was able to upregulate osteogenic and adipogenic, but not chondrogenic, transcription factors. Interestingly, MSC osteogenesis was enhanced following IL-22 exposure, while the combination of IFN-γ and TNFα with or without IL-22 suppressed MSC osteogenesis [13]. This evidence could suggest a scenario in which biomechanical stress through the induction of IL-23 and, consequently, IL-22 expression could support the proliferation of MSCs, giving them an osteogenetic potential.

Given the strong genetic association between SpA susceptibility and HLA-B27, it is fascinating to speculate that the presence of HLA-B27 may affect the function of MSCs in patients with AxSpA. Liu and co-workers in a recent work [14] used MSCs obtained from inflamed enthesis of patients with AS and spinal ankylosis to demonstrate that HLA-B27-dependent activation of the sXBP1/RARB axis/TNAP accelerated the mineralization of AS-MSCs. Furthermore, the implantation of MSCs obtained from patients with AS in the lumbar spine of NOD-SCID mice was able to reproduce the pathological bone apposition of AS.TNAP inhibitors, including levamisole and pamidronate, were able to inhibit in vitro the mineralization of MSCs obtained from patients with AS and block bone apposition in vivo [14].

Beyond the ability of MSCs to participate in bone remodeling through differentiation into osteoblasts, they also act by indirectly regulating osteoclastogenesis. In a recent study, AS-derived MSC or MSC from healthy donors (HD-MSC) were co-cultured with CD14+ monocytes in an osteoclast induction medium [15]. AS-MSCs showed a greater ability to inhibit osteoclastogenesis than HD-MSCs. This process appears to be mediated by an over-production of CXCL5 by the AS-MSCs, which inhibits osteoclastogenesis. CXCL5 hyper-expression appears to be regulated by miR-4284, which is found to be hypo-expressed in AS-MSCs [15].

Although all these studies support a pathogenic role of BM-MSC in AS, experimental data indicate that infusion of umbilical MSC can alleviate disease activity and symptoms in patients with SpA [16]. In light of previous comments, it is possible to imagine that genetic factors such as HLA-B27 can induce a pro-inflammatory and pro-osteogenetic phenotype at the BM-MSC of patients with SpA, which would not happen in MSCs obtained from healthy donors. Further studies are needed to investigate several features of MSC therapy, such as cell origin, dosage, and disease stage most appropriate for an ideal intervention.

### Fibroblast-like synovial cells

Fibroblast-like synovial cells (FLS) are not simple bystanders in the inflammatory process observed in inflammatory arthritis. Particularly in rheumatoid arthritis, FLS are aberrantly activated and participate directly to the synovial hyperplasia and indirectly via strict bidirectional crosstalk with the immune infiltrating cells and secretion of cytokines and proteases [17].

In SpA, FLS are cells of major interest also for their participation in the aberrant bone remodeling typical of the SpA spectrum [18]. Although the etiology of aberrant bone formation in SpA is not fully known, FLS can differentiate and act as precursor of osteoblast-like cells contributing to ectopically bone formation on bone, entheses, and tendons [19]. Pro-inflammatory cytokines such as TNFα [19], IL-17 [20], and IL-9 [21] are known to influence their inflammatory phenotype and osteogenic potential. However, other evidence suggest that in SpA, FLS participation in bone remodeling is mostly independent from the inflammatory environment and seems rather caused by an intrinsic transcriptional signature [22]. This may be one of the explanations of the relatively poor efficacy of old and new therapies in halting the bone remodeling associated to SpA. The theory dates back to the early 2000s and is supported by identifying a SpA associated synovial signature that was not pointing to immune or inflammatory pathways but rather to muscle-/myofibroblast-associated pathways [22]. In line with this observation, other reports suggest that the RANK/RANKL/Osteoprotegerin axis in SpA FLS is partially disconnected from the systemic and local inflammation and is not responsive to anti-TNFα treatment [23]. FLSs also indirectly contribute to ossification by producing IL-26 that, enriched in SpA synovial fluid and axial faced joints, contributed to increased bone mineralization in human osteoblasts [24].

A unifying approach would consider inflammation, mechanical stress, and an intrinsic propensity of FLS to osteogenesis as major determinants in the bone formation in SpA.

Therefore, the FLS are not new cellular players in SpA; however, it is relevant to mention a change in perspective and the development of new approaches targeting the FLS directly and not only the surrounding inflammatory environment. Recently, new evidence has been obtained in clearly fibrotic conditions such as idiopathic pulmonary fibrosis, which have been translated to SpA [25]. Stougaard et al. tested the effect of the antifibrotic approved drug pirfenidone on SpA FLS. In culture, pirfenidone inhibited FLS proliferation and expression of myofibroblast markers. Interestingly, pirfenidone acted also inhibiting the mineralization by osteoblast [25]. To date, the data are of unproven clinical significance; however, they offer an example of alternative therapeutic strategies for SpA.

Very recently, data on the use of the mTOR inhibitor, rapamycin, in the HLA-B27 transgenic rat model of SpA have been published [26]. mTOR inhibition reduced the incidence and severity of arthritis and spondylitis and blocked the associated new bone formation and erosion. Rapamycin exerted an anti-inflammatory effect on PBMC and dampened the osteogenic properties of FLS independently of IL-17A and TNFα cytokines [26].

## Adaptive immunity

Adaptive immune cells, as a consequence of clonal recognition of non-self (foreign) antigens, confer life-long protection against a vast number of challenges and orchestrate the response of the entire immune system. T lymphocytes, primed in the thymus and characterized by the expression of the costimulatory molecule CD4 or CD8 on their surface, are the cornerstones of adaptive immunity. The recognition of protein antigens mediated by the specific T cell receptor expressed on their surface is the first of a series of events that shape the organized response to external challenges. When a self-antigen is erroneously recognized, or when the activation mechanisms are altered or dysregulated, T cells can become pathogenic, as in inflammatory arthritides. We will describe the efforts to understand antigen-driven responses in AS patients and then enumerate the various CD4 and CD8 cell phenotypes linked to SpA pathogenesis.

### Antigen specificity

Soon after discovering the strong association of AS with HLA-B27, a common HLA class I allele, it was hypothesized that the physiologic function of HLA-B (that is presenting intracellular peptides to CD8+ T cells) would be the key to explaining the AS pathogenesis. The search for an autoantigen, for a microbial antigen exhibiting a molecular mimicry or, at least, for HLA-B27-specific CD8+ T lymphocytes, has kept researchers active for many years, with mixed success. Using structural biology and proteomics approaches, several authors have tried to identify an arthritogenic peptide able to induce CD8+ self-reactive responses [27], but no decisive evidence supporting this theory has been found. A parallel argument for conventional antigen presentation by HLA-B27 as a key causative event is the association of ERAP1 polymorphisms with AS, interestingly seen only in the context of HLA-B27. The ERAP1 peptide is an aminopeptidase involved in MHC class I peptide processing [28]. It has been proposed that gene variants of ERAP1 could affect the interaction of HLA-B27 with peptides.

Expanded oligoclonal T cell populations have been described in patients AS (and other SpA conditions), almost exclusively in the synovium [29, 30], suggesting an antigen-driven expansion likely influenced by the environment. More recently, a number of TCR motifs reactive to HLA-B27-peptide complex in AS patients’ blood were identified [31]. These were prevalently in the CD8+ compartment, although the CD4+ TCR repertoire also appeared altered in AS patients compared to a healthy cohort [32].

Indirect evidence of adaptive immunity processes in SpA is also provided by the microscopical analysis of the SpA synovium [33], which shows T lymphocytes often organizing in aggregates, and in certain patients, in B cell rich follicles, arranged into structures similar to germinal centers. The role of these structures is not clear, nor the events that lead to their formation, although they could be the product of cell recruitment secondary to preexisting, antigen-independent, inflammatory processes.

### CD4^+^ cells

T helper cells, characterized by CD4 expression, after the recognition of the antigen and the TCR engagement signal, integrate stimuli leading to cell differentiation. The induction of the dominant transcription factor then controls the gene program, which includes specific cytokine production and molecules that mediate trafficking to target organs. Dysregulated T cell commitment mechanisms can contribute to pathogenic responses, such as autoimmunity mediated by Th1 and Th17 cells. An important T helper subset, Tregs, is specialized in terminating the host immune response, and novel T helper cell subsets, more recently proposed, such as Th9 and Th22, have a role in mucosal immunity. The possible contribution of each T helper cell subset to the pathogenesis of SpA is discussed below.

#### Th1

T helper 1 cells (Th1) are characterized by the release of interferon-gamma (IFN-gamma) and TNFα. They mediate cellular immune responses and, as a result of the release of inflammatory cytokines, activate other immune cells. The existence of a disease-specific role for IFN-γ in AS is debated. No significant differences in the percentage of Th1 cells were found by Szanto et al. [34], while another group described an increase of IFN-γ-producing cells in AS peripheral blood, with a reversal to normal levels after anti-TNFα treatment [35]. IFN-γ is also produced by Th17 cells, more specifically by a subset termed Th17.1 or Th17 (exTh1), found accumulating in the rheumatoid [36] and the psoriatic arthritis (PsA) synovium [37].

#### Th17

Of all the T helper subsets, the most substantial evidence for a central role in SpA pathogenesis converge on Th17. Numerous genetic and immunological studies suggest a Th17 dysregulation could be critical in the development of SpA, often considered “type 17” (or “type 3”)-driven inflammatory diseases. Currently, targeting type 17 responses with monoclonal antibodies is indeed one of the most effective therapeutic approaches for AS and PsA. Although the percentages of IL-17-positive CD4+ T cells and IL-22-positive CD4+ T cells are increased in the PBMCs of both patients with AS and PsA compared with healthy control subjects [38, 39], suggesting a direct pathogenic role, the physiological function of Th17 cells consists of defense against extracellular bacteria and fungi. The differentiation of the naïve CD4+ cell into Th17 is driven by a combination of cytokines, such as IL-1β, IL-6, TGFβ, and IL-23, as identified by in vitro experiments [40]. In AS, cells and molecular events predisposing to an exaggerated type 17 responses have been described in the gut (IL-23-producing resident macrophages [41], dysbiosis, and autophagy [42]) and in the enthesis [43, 44]. These can all contribute to the inflammatory milieu that expands Th17 cells in vivo. The exact events that render physiological anti-microbial Th17 pathogenic cells are still not clear but might involve genetic variants controlling Th17 recruitment and maturation. One feature of pathogenicity described so far is the co-expression of inflammatory cytokines such as IFN-γ and GM-CSF [45]. Th17 cells able to produce multiple inflammatory cytokines have been described at inflammatory sites in SpA: the Th17 heterogeneity is an argument for treating SpA patients with molecules able to simultaneously target several Th17-associated inflammatory cytokines [46, 47]. A deeper characterization of the functional range of Th17 cells, especially at the inflammatory sites, is warranted.

#### Th9

Th9 T cells, characterized by IL-9 production, are among the most recent additions to the T helper differentiation paradigm. Previously identified in the psoriatic skin [48], in SpA, they have been identified in the gut of PsA patients [49], where IL-9 is produced to specialized T helper cells and Paneth cells, in association with the antibacterial α-defensin 5, whose production is further reinforced by an IL-9 mediated autocrine loop, demonstrating interesting crosstalk between adaptive and innate mucosal responses. In the same patients, a Th9 signature was also found in the synovium and peripheral blood, where some of these cells expressed intestinal homing receptors.

#### Th22

Th22 cells are often considered a subdivision of Th17 because the signature cytokine, IL-22, is often found produced by Th17 cells. The transcriptional regulation of IL-22 is somewhat separated, being controlled by the aryl hydrocarbon receptor, a ligand-induced transcription factor [50].

IL-22 is part of the IL-10 family and it is elevated in several immune-mediated diseases, including AS [51]. IL-22 has a strong association with gut immunity, with its effect in regulating mucosal integrity and clearance of intestinal pathogens [52]. The discovery in GWAS of disease associations with IL-22 signaling, as well as preclinical models, has led some investigators to hypothesize a pathogenic role for IL-22 in IBD [52]. Although elevated in the synovial fluid of PsA patients [53], a clear mechanistic contribution to musculoskeletal manifestations has been characterized in only one study. Starting from the known effect on liver, gut, and skin stem cells [54], El-Zayadi et al. [13] proposed that IL-22 could drive MSC osteogenesis, the biological phenomenon mediating the process of new bone formation in SpA, which might happen independently and in parallel. A role for Th22 cells in the initiation of the disease has not been found yet, but their involvement in mucosal immunity and their putative effect on bone metabolism make the study of Th22 an attractive pathway for future research.

#### Tregs

In the best-studied SpA experimental model, the B27-TG rat, a defect of regulatory T cells was observed: Tregs were there characterized by a higher IL-17/IL-10 ratio [55]. In humans, there is no overall consensus regarding Tregs in the peripheral circulation, and a recent metanalysis [56] noted how, in the various reports published so far in AS, different flow cytometric parameters have been used to define Tregs. In the SF of AS patients, a significant increase in Tregs was found compared with peripheral blood [57], potentially indicating Tregs’ recruitment to this site of inflammation. Synovial Tregs preserved their suppressive activity and effectively inhibited the proliferation of effector T cells [58]. Little is known about the alteration of Tregs in the SF and tissue during a SpA flare, i.e., if the tissue microenvironment or soluble inflammatory mediators can influence their phenotype and functionality. In a study focusing on another organ that can be affected in AS, the terminal ileum, a high number of Tregs were identified [59], the majority of which were able to produce IL-10, suggestive of a putative role in the regulation of intestinal inflammation and in particular of Th17 responses, similar to a well-characterized regulatory mechanism in murine colitis [60]. Studies reporting on Tregs in inflammatory arthritic conditions focus on blood rather than tissue sites, where the local inflammatory microenvironment might alter the gene expression, post-transcriptional regulation, and consequently the function, limiting our understanding of the actual role of Tregs in these conditions.

### CD8+ cells

The pathogenic role of CD8^+^ T cells in initiating AS was reconsidered with the evidence that the HLA-B27 transgenic rats develop the disease in the absence of CD8^+^ cells [61]. Recent years have again seen a renovated interest in CD8^+^ T cell research in AS. Genetic studies have linked AS to new CD8+ associated factors, such as *TBX21*, *EOMES*, and *RUNX3* [62], leading to a new research path, including emerging CD8^+^ subsets discussed below.

#### Tissue-resident memory cells

Recently identified, tissue-resident memory (TRM) cells colonize tissues without recirculating, providing prompt in situ response against infections. Because absent in the circulation and consequently missed by PBMC phenotyping, their transcriptional and functional profile has been characterized only recently, and they are attracting growing interest as possible key players in tissue immunopathology and autoimmunity. One of their features, especially at barrier sites, is integrins expression, transmembrane receptors that mediate the cell-to-matrix adhesion. The upregulation of α4β7 integrin on the cell surface is a step that facilitates entrance to the gut [63], which is later lost in favor of αEβ7 pairing, that together with the expression of CD69, granzyme B, and the downregulation of Ly6C and CD122 expression, permit their stability in the tissue [64]. Initial evidence suggested a contribution of TRM to Crohn’s disease [65]. In AS, TRM-like cells have been identified in the SF [66]: these CD8+ cells expressing integrin, in fact, presented a distinct transcriptional profile suggesting a gut origin. Cells with similar profiles were consequently confirmed in the gut, peripheral blood, and synovia of patients with AS [67], suggesting the potential to recirculate in the AS target organs, providing indirect evidence of a gut-joint migration axis. What is TRM’s actual pathogenic role in AS and what messages induce trafficking to the synovial environment remain unclear, yet this mechanism could help clarify the association between gut inflammation and musculoskeletal.

#### Cytotoxic CD8+ and Tc17 CD8+

One of the main functions of CD8+ T cells, together with producing inflammatory cytokines, particularly TNFα, IFN-γ, and IL-17, is target cell killing, both by secreting perforin/granzyme (peptides able to induce apoptosis) or signaling through Fas/FasL pathway. A recent study showed an altered cytotoxic gene and protein activity in the peripheral blood of AS patients but elevated in the SF compared to rheumatoid arthritis and osteoarthritis [68].

The expansion of type 3 immunity, characterized by IL-17A production, in SpA includes CD8+ cells. IL-17-producing CD8+ (Tc17) has been characterized in PsA patients’ SF [69] and, previously, in a brief report, observed in AS [70].

## Innate and innate-like immune cells

### Type 3 innate lymphoid cells

ILC are a group of immune cells developing from a common lymphoid progenitor but devoid of any typical myeloid, T and B markers. Transcriptomic and flow cytometry studies identified at least three functional groups—ILC1, ILC2, and ILC3—each one featuring a distinct cytokine repertoire. Specifically, ILC3 are mucosal-restricted cells participating to the type 3 immune response toward extracellular fungi and bacteria, mainly producing IL-17 and IL-22 in response to IL-23 stimulation as reviewed elsewhere [71]. In line with ILC3 primary localization, the gut is recognized as one of the main sites of activation and priming [71]. ILC3 are significantly expanded in the gut of both SpA patients showing subclinical gut inflammation and in patients affected by inflammatory bowel disease [72, 73]. The expansion of intestinal ILC3 seems to be driven by dysbiosis as it correlates with the bacterial epithelial infiltration and induced by the secretion of IL-23 by CXCR1^+^CD59^+^ macrophages [41, 74]. In light of the role played by IL-17 in maintaining the intestinal epithelial barrier integrity [75], it is not clear whether ILC3s have a direct pathogenic role or represent rather an attempt to resolve gut inflammation. We and others demonstrated that ILC3 gain migratory properties, recirculating from the gut as are expanded in blood, bone marrow, and synovial fluid of patients with AS, where it correlates with the Bath Ankylosing Spondylitis Disease Activity Index [72]. Similar observations have been made in PsA [76], and also in healthy controls ILC3 seems to have a preferential entheseal localization compared to blood [77]. The mucosal origin is supported by the expression of the intestinal homing integrin α4β7 by recirculating ILC3 and the upregulation of its receptor MAdCAM1 in the bone marrow of AS [72]. Therefore, it seems likely that ILC3 act as a shuttle transferring the inflammation and type 3 immunity, including IL-17 or IL-22 production from intestinal lymphoid rich cryptopatches to the tissues affected by SpA, namely, entheses, synovium, and bone marrow [78–80].

### Mucosal-associated invariant T cells

Mucosal-associated invariant T (MAIT) cells are a distinct population of RORγt^+^CD3^+^CD4^−^CD8^±^ T cells with a predominant innate-like behavior. Despite responding to the activation of the semi-invariant TCR receptor restricted to Major Histocompatibility Complex (MHC) class I-like molecule MR1, MAIT cells exert a relevant MR1-independent innate-like activity in response to inflammatory cytokines and bacterial metabolites [81]. Following activation, MAIT cells secrete type 3 inflammatory cytokines such as IL-17 and IL-22 [82]. MAITs are recognized as a relevant source of IL-17 in SpA spectrum. Despite some discrepancies, multiples studies have demonstrated alteration in MAIT composition in SpA. Circulating MAIT cells are reduced in AS compared to healthy controls with a relative increase in the percentage of IL-17-producing cells and a correlation between CD69 expression and the disease activity score [82]–[84]; Gracey et al. demonstrated that this reduction is paralleled with an increased frequency of MAIT cells in SF where they are hyperresponsive to IL-7 leading to abundant IL-23 independent IL-17 production [83]. These data suggest the recruitment of MAIT cells from the circulation to the site of disease in SpA. Moreover, the response to IL-7 stimulation has been proposed as a possible link with SpA pathogenesis for both the genetic contribution due to the association of IL7R polymorphism to AS and the mechanical stress that acts as stimulus for IL-7 secretion by synovial fibroblasts [85]. Interestingly similar observations come from PsA, where IL-17-producing MAIT cells are enriched in SF and are responsive to IL-23 stimulation [86].

### γδ T cells

γδ T cells are atypical T cells developing from a common lymphoid progenitor and functionally committed in the thymus [87]. The peculiarity of the γδ T cells is the expression of an oligoclonal TCR constituted by gamma and delta chains that is not restricted to MHC and can respond to non-peptidic molecules [88]. The prompt TCR-independent response with a type 1 and type 3 cytokines secretion, including IL-17 and IL-22, confers them an innate-like behavior [89]. In normal conditions, they can be found in rodents and in humans predominantly at the epithelial and mucosal surfaces, both common sites of inflammation in SpA, and at a lower frequency in circulation [90]. The prompt and abundant secretion of IL-17 makes these cells candidate pathogenic effectors in the context of SpA [89]. Studies investigating the frequency and role of γδ T cells in inflammatory rheumatic disease date back to their first identification. The investigations on the frequency of circulating γδ T cells in SpA often produced conflicting results; however, IL-17-producing γδ T cells were found increased in the synovial fluid of human AS and SpA spectrum [82, 91]. In this context, IL-23 was identified as the main inducer of γδ T cell activation and IL-17 production [91]; in addition, we recently identified the ability of IL-9 to induce IL-17 production in PsA synovial fluid by γδ T cells [92].

The primarily mucosal localization raises the question about the possible migration of γδ T cells from the barrier sites to joints and entheses. However, γδ T cells have been found to be abundant in normal entheses in both mice and human exhibiting a distinct transcriptional phenotype compared to blood, suggesting γδ T cells as entheseal resident cells [77, 93]. Overexpression of IL-23 in mice led to activation and expansion of IL-23R^+^γδ T cells, making them the main producers of IL-17 within tissues exposed to mechanical stress (e.g., enthesis, aortic root, ciliary body) [94]. In normal human spinal entheses, however, IL-23 was not the main driver of IL-17 production from γδ T cells [93]. Further studies on human SpA tissue are warranted to characterize the γδ T cell biology more fully in these conditions.

### Mast cells

Although mast cells have been well characterized in other conditions such as skin psoriasis and were known to infiltrate PsA synovium since the early 80s, their relevance in SpA has been rediscovered only recently [95]. Mast cells are known to participate in RA synovitis [96]; however, Baeten and colleagues demonstrated a preferential enrichment in mast cells in PsA synovium compared to RA, and surprisingly mast cells were the most abundant IL-17^+^ cells representing 63% of IL-17^+^ synovial cells [97]. Similar observations have been made in axial SpA by analyzing the facet joints of late-stage AS patients, where mast cell were the second most abundant IL-17^+^ cells after neutrophils [98].

These studies generated high scientific interest pointing to the mast cells as a newly identified producer of IL-17; however, later studies failed to demonstrate the active production of IL-17^+^ by mast cells that seem to lack the transcriptional program for IL-17 production [99]. In fact, recent work demonstrated that mast cells act as a buffer for IL-17 by mediating internalization of exogenous IL-17, its storage, and the prompt release after stimulation [99]. Interestingly, mast cells were the most abundant IL-17^+^ cell also in unaffected tissue, including skin and gut; by contrast, a reduction in IL-17^+^ mast cells was observed in inflamed IBD intestinal mucosa [100]. These observations are not enough to clearly establish a primary role for mast cells in SpA, whether they act primarily as scavengers for the excess of IL-17 dampening the inflammation or contribute to the inflammation by releasing inflammatory cytokines including IL-17 or both activities.

Very recently, in an animal model of SpA, mast cells have been demonstrated to be increased in sacroiliac joints and in the intestine, where they demonstrate an activated phenotype and serve as a relevant source of GM-CSF contributing to the disease manifestations [101]. In the same model, the mast cells stabilized cromolyn, with benefits on both disease manifestation and immune cell infiltration in the target tissue [101]. Conversely, mast cells therapeutic targeting of mast cells in human SpA using nilotinib showed some efficacy only on peripheral SpA [102]. More extensive studies and new mast cell-targeting strategies are warranted to establish the role of mast cells in SpA definitively.

### Neutrophils

Neutrophils are one of the early tissue invaders virtually in any site of infection and inflammation. Neutrophil infiltration has been classically observed in the course of arthritis. However, multiple reports observed a preferential neutrophil involvement in PsA compared to RA, and both TNFα and IL-17 are known chemoattractants for neutrophils [103, 104].

Similar to mast cells, studies on facet joints of AS patients demonstrate that the majority of IL-17+ cells were neutrophils [98]; a similar infiltration of IL-17^+^ neutrophils has been reported in PsA synovium, psoriatic skin [6, 105].

However, the same work failed to demonstrate whether IL17 was produced due to autocrine secretion or whether the positivity was due to the binding of IL-17 to the IL-17+ receptor expressed on neutrophils [98]. In recent functional experiments, in fact, neutrophils were unable to produce IL-17 [106]. A buffering mechanism similar to that observed for mast cells could be therefore postulated. In line with this consideration, one of the ways by which neutrophils promptly release the IL-17 in an inflammatory fashion is NETosis, previously observed in psoriasis and more recently reported to be exaggerated in radiographic axial SpA patients [107, 108].

Very recently, Boutet and colleagues explored the role of the neutrophil activated cytokine IL-36, an under investigated member of the IL-1 family, in the pathogenesis and treatment response in PsA [104]. To note, bulk RNA sequencing performed on early treatment naïve PsA and RA synovial tissue demonstrated a strong neutrophil signature in PsA tissues, which was consistent with immunohistochemical staining for neutrophil-derived proteins. In the same cohort, it was observed that there was an imbalance between IL-36 agonists and antagonists that correlated with a poorer response to DMARDs [104].

This and other works suggest the existence of multiple mechanisms by which neutrophils could contribute directly and indirectly to SpA pathogenesis that deserves to be further investigated in the near future.

### Invariant natural killer T cells

Invariant natural killer T cells (iNKT) is another innate-like T cell type recently identified as a candidate cellular player in SpA [6]. Similar to MAIT cells, despite being formally designated T cells, iNKT express a semi-invariant TCR consisting of an invariant α chain and a restricted β chain repertoire. However, the iNKT activation and antigen recognition are mediated by the MHC-like receptor CD1d in a TCR dependent and independent fashion, conferring an innate-like behavior and causing the prompt secretion of a significant amount of cytokines [6]. Similar to T helper cells, iNKT show some degree of plasticity, and on the basis of their transcriptional profile and cytokine repertoire, at least three phenotypes have been described: iNKT1, iNKT2, and iNKT17, analogous to Th1, Th2, and Th17, respectively [109]. In the SKG mice model of SpA, iNKTs were skewed toward IFN-γ-producing iNKT1 that seems to ameliorate arthritis [110]. In RA SF, a reduced number of iNKT cells and IFN-γ production could possibly contribute to arthritis persistence [110]. Similarly, in TNFα (DeltaARE/+), the genetic ablation of iNKT function was associated to a more severe joint and gut inflammation, and the activation of iNKT seems to be influenced by the crosstalk with dendritic cells chronically stimulated by TNFα [111]. Consistently, most of the reports coming from inflammatory bowel disease patients [112] report a reduction in iNKT in line with the protective role observed in the animal models.

The relevance of iNKT cells in human SpA remains incompletely understood. However, very recently, Elewaut’s group identified a novel RORγt^+^T-bet^lo^PLZF^−^ iNKT circulating in healthy individuals and significantly expanded in inflamed joints [113]. These cells have a Th-17-like phenotype being responsive to IL-23 and producing IL-17. In SpA patients, the frequency of iNKT cells in circulation was reduced and, by contrast, was increased in synovial fluid [113]. Notably, among the synovial fluid iNKT cells a significant proportion were IL-23R^+^. Ex vivo experiments confirmed that iNKT and γδ T cells, although not the most abundant cells, are a major source of IL-17, IL-22, and INFγ in SpA joints [113]. From a therapeutic perspective, the same group demonstrates a selective impairment of IL-17 production by testing a small molecule mediated RORγt inhibition, preserving the production of IL-22 [113]. These data seem very intriguing from both a pathogenic and therapeutic perspective, although the block of RORγt is, for obvious reasons, not specific for iNKT, but has a rather broad activity toward many cellular players involved in type 3 immunity.

## Conclusions

The pathogenesis of AS is multifactorial and involves a complex interplay between innate and adaptive immunity and non-immune stromal cells. Recent advances have identified a myriad of novel cellular players that can contribute to the pathogenesis of SpA. Although it is currently impossible to draw a cellular hierarchy, autoinflammatory stimuli, possibly originating at the enthesis or the intestine, both anatomical sites that are exposed to barrier challenges or biomechanical factors, trigger innate immunity responses. In contrast, adaptive immune cells and processes are involved in the perpetuation of inflammation and damage. Numerous outstanding questions remain, including the role of stromal cells in influencing the tissue immune landscape, the relative contribution of innate and adaptive immunity cells to the overexpression of type 17 immunity observed in SpA, and the composition of the intricated network that drives pathogenic IL-17-mediated responses.
